# Prevalence of Sepsis Continuum in Patients With Type 2 Diabetes Mellitus at Tupua Tamasese Meaole Hospital in Samoa

**DOI:** 10.7759/cureus.17704

**Published:** 2021-09-04

**Authors:** Stacey S D'Almeida, Rustum M Moodley, Viali Lameko, Randell Brown

**Affiliations:** 1 Medicine, Oceania University of Medicine, Apia, WSM; 2 Internal Medicine, Oceania University of Medicine, Apia, WSM; 3 Research, Oceania University of Medicine, New York, USA

**Keywords:** samoa, traditional medicine, sepsis, internal medicine, diabetes

## Abstract

Background

Individuals with longstanding type 2 diabetes mellitus (T2DM) have a significantly higher risk for infection caused by immune dysfunction, resulting in sepsis continuum (sepsis, severe sepsis, and septic shock) if not adequately addressed. In sepsis, organ dysfunction occurs because the host’s response to infection is impaired, more so in severe sepsis. In septic shock, persistent hypotension happens, requiring vasopressors despite aggressive fluid management. The internal medicine (IM) ward plays a critical part in managing patients with sepsis. However, the prevalence of sepsis has been investigated extensively in an intensive care unit (ICU) setting instead of the IM ward. This study aimed to determine the prevalence rates of sepsis, severe sepsis, and septic shock in patients with T2DM admitted at an IM ward in Samoa.

Methods

This retrospective hospital record-based study was conducted over four months on 100 patients with T2DM admitted to the IM ward within the sepsis continuum. Participants were selected by convenience sampling, and the diagnosis was determined from the admission notes.

Results

The prevalence rates of sepsis, severe sepsis, and septic shock in patients with T2DM admitted to the IM ward were 80%, 12%, and 8%, respectively.

Conclusion

The most frequent presentation in individuals with T2DM who are within the sepsis continuum upon admission to the IM ward was sepsis, followed by severe sepsis and septic shock.

## Introduction

Diabetes mellitus (DM) is characterized by elevated glucose levels caused by either a defect in insulin secretion from the pancreas or peripheral resistance to insulin effects. Patients with DM are generally more prone to infections resulting from immune dysfunction, hyperglycemia, and micro- and macrovascular complications [1]. The scale of sepsis includes worsening of illness and inflammation, starting from sepsis, followed by severe sepsis and finally, septic shock. Sepsis is classified as an increased host reaction to infection, which is verified by positive cultures for organisms at the site, causing organ dysfunction. In severe sepsis, an individual’s condition further advances, accompanied with organ deterioration involving the kidneys, lungs, and/or the gastrointestinal system. Individuals at this phase develop organ hypoperfusion symptoms, such as oliguria and altered mentation [2]. In septic shock, intense circulatory, metabolic, and cellular breakdowns take place, resulting in amplified mortality compared with sepsis. At this level, hypotension is pervasive, requiring vasopressors to sustain a mean arterial pressure of >65 mmHg, regardless of having acceptable fluid resuscitation. In addition, the blood lactate level is >2 mmol/L because of reduced end-organ perfusion [3]. Clinically, altered mentation, oliguria, persistent hypotension, and lactic acidosis also develop.

Sepsis is diagnosed clinically at bedside using the systemic inflammatory response syndrome (SIRS) criteria or the quick Sepsis-related Organ Failure Assessment (qSOFA) score plus the procalcitonin level [4]. The SIRS criteria involve four parameters: respiratory rate (>20 breaths per minute), heart rate (>90 beats per minute), temperature (>38°C or <36°C), and white blood cell count (<4000 or >12,000 cells/mm3). Scores of >2 indicate sepsis. The presence of a tested infectious source also confirms sepsis [2]. The qSOFA score has three criteria: altered mentation, reduced systolic blood pressure (<100 mmHg), and high respiratory rate (>22 breaths per minute). Scores of >2 indicate a high risk for in-hospital mortality [3]. Procalcitonin is a biomarker of sepsis; levels of >1 ng/ml strongly indicate bacterial sepsis [5], thereby valuable for guiding antimicrobial treatment. The most typical places of infection include the gastrointestinal, respiratory, genitourinary, skin, and soft tissue. In individuals with type 2 DM (T2DM), pneumonia is the most common clinical diagnosis causing sepsis [6].

In 2017, roughly 48.9 million sepsis cases were reported globally [7]. Of these cases, 11 million died, accounting for 19.7% of deaths worldwide. Approximately one-half of the overall sepsis-related deaths emerge from noncommunicable diseases such as DM and an underlying injury. In Australia, the annual sepsis mortality rate is approximately >5000 people, with a disproportionate number of individuals being of Aboriginal and Torres Strait Island ethnic origin [8]. These individuals, who live in remote areas, have an above-average rate of noncommunicable diseases. The Pacific Islands have a substantial proportion of people suffering from DM. Of the 10 nations with the most sizable DM prevalence globally, six are within the Pacific [9]. These parties have a greater risk for DM sequelae, including sepsis. A study in Tonga reported that the levels of DM-associated ailment and mortality have increased, thereby detailing the requirement for improved DM care [10]. Similar to other Pacific Island countries, Samoa has an increased number of T2DM cases, particularly in individuals aged 25-74 years across both sexes [11]. In Samoa, 1500 individuals are admitted yearly to the internal medicine (IM) ward, and a quarter of them reportedly have DM. In addition, two-fifths of these DM admissions have sepsis [12]. The increasing rate of DM in the Pacific and the lack of adequate monitoring by medical practitioners of patients once they are diagnosed could increase the likelihood of infection and presentation along the sepsis continuum. Samoa is a developing country; therefore, uncontrolled DM and late presentation of sepsis along the sepsis continuum can cause undue strain on the healthcare system.

The sepsis continuum has a considerable glycemic variability. Blood glucose levels tend to increase along the sepsis continuum [13]. The long-term glycemic control is generally evaluated by the hemoglobin A1c level (HbA1c), which measures glycemic control over the last three months. The HbA1c test has an independent predictive value for mortality and hospitalization duration in individuals with sepsis [14]. However, the linkage between HbA1c and the sepsis continuum remains unreported.

Furthermore, reports on the relationship between sexes in individuals with sepsis are inconsistent. On average, sepsis develops more in males than in females regardless of age. In fact, older male individuals are more inclined to developing severe sepsis [15]. However, the association between sex and age across the sepsis continuum has not yet been reported in Samoa.

In most studies, data regarding the epidemiology, etiology, and management of individuals with sepsis are mainly obtained from intensive care units (ICU) in high-income nations. The statistics attained from studies using ICU data have primarily influenced the current guidelines on sepsis protocol [16]. In low-income nations, such as the Pacific Islands, ICU facilities are scarce. Therefore, information necessary for guiding healthcare policies and procedures is insufficient in Pacific Islands. Individuals who have a higher risk for sepsis, such as those with advanced age and/or comorbidities, including DM, are more commonly hospitalized outside the ICU setting, mainly the IM wards [17]. The apparent mortality rates for sepsis, severe sepsis, and septic shock are 25%-30%, 40%, and 70%, respectively [18]. When afflicted with ailments, people living in the Pacific Islands frequently utilize both traditional and western medicines. According to a study in Tonga, a diabetic wound treated with traditional medicine would develop sepsis more often and require amputation [19], implicating that a person with diabetic sepsis must be treated promptly. The delay of required medical treatment may result in infections along the sepsis continuum. However, the relationship between traditional medicine usage and the delay of medical therapy to diabetic sepsis remains unexplored in Samoa.

Appropriate counseling and stringent medical follow-up can help identify high-risk patients with diabetes to minimize these life-threatening complications. Unfortunately, evidence regarding the sepsis continuum and the prevalence of this complication remain undetermined in Samoa. Hence, this study aimed to assess the sepsis data in the largest hospital in this country. Our main objective was to identify the prevalence rate of sepsis, severe sepsis, and septic shock in patients with T2DM admitted to the IM ward. Next was to identify the clinical and demographic characteristics of these patients. Lastly, we sought to record the number of patients in each category along the sepsis continuum who consulted a traditional healer before being admitted to the IM ward.

## Materials and methods

This retrospective hospital record-based study was conducted in the IM ward of Tupua Tamasese Meaole (TTM) hospital in Apia, Samoa, between February and May of 2020. Through convenience sampling, we retrospectively selected patients recorded in the inpatient registry book of the IM ward. We found 590 patients admitted to the IM ward, and 215 of them had T2DM. Of these 215 patients, the initial 100 patients were sampled from the inpatient book, and we determined whether they met the inclusion criteria. The individual clinical files of these 100 patients were subsequently recovered from the records department of TTM hospital.

The inclusion criteria were as follows: either Samoan male or female with T2DM, 45-80 years old, diagnosis of T2DM for ≥5 years, more than one DM medication (e.g., metformin or glipizide), and a recorded diagnosis along the sepsis continuum in their admission notes. Conversely, the exclusion criteria were the following: T2DM for <5 years and not being of Samoan ethnic origin. To specify the prevalence rates according to sepsis subcategories, we classified the participants into sepsis, severe sepsis, or septic shock, as documented in their respective clinical files on admission. The diagnosis of sepsis was made by physicians in Samoa in accordance with the sepsis guidelines [3]. We recorded the sex and age of these participants and tabulated the kind of infection they had upon admission (e.g., respiratory, urinary tract infection, and skin/soft tissue infections). Laboratory findings such as HbA1c, random blood glucose (RBG), and white blood cell count were also collected upon admission. Before presentation at the IM ward, the usage of traditional medicine was identified from the admission notes. The institutional review board of the Oceania University of Medicine and the National Health Service of Samoa approved this study.

Descriptive statistical analyses were calculated, and variables are expressed as percentages, medians and interquartile ranges (IQR), and means and standard deviations (SD). The prevalence rate was also quantified. We used the chi-square test to compare the categorical variables and to gauge the linkage between sepsis status and sex. The normality of data distribution for continuous variables such as median age, mean RBG, and mean HbA1c were ascertained using the D’Agostino-Pearson normality test. These variables did not follow a normally distributed curve; thus, their significance was examined by the Kruskal-Wallis test (nonparametric). The Kruskal-Wallis test was also employed to analyze whether a significant relationship existed in the underlying age distributions of patients with sepsis, severe sepsis, or septic shock and the connection of RBG and HbA1c to the sepsis continuum. Further, p < 0.05 was deemed to be statistically significant. Analyses and verification were executed using Microsoft Excel, SPSS version 27, and Graph Pad Prism version 8.4.3.

## Results

The prevalence rates of sepsis, severe sepsis, and septic shock in patients with T2DM admitted to the IM ward were 80%, 12%, and 8%, respectively. Female patients (52%) were slightly more than the male patients (48%). Out of 100 patients, 11 (11%) used traditional medicine. The median age of all included patients was 59 (IQR, 45-79 years). Table 1 summarizes such details.

**Table 1:** Demographic and sepsis presentation summary of adults with type 2 diabetes mellitus admitted at Tupua Tamasese Meaole Hospital between February and May of 2020. IQR: interquartile range.

Total number of patients (n)	100
Males	48 (48%)
Females	52 (52%)
Patients with sepsis	80 (80%)
Patients with severe sepsis	12 (12%)
Patients with septic shock	8 (8%)
Patients using traditional medicine	11 (11%)
Median age of all participants (IQR)	59 (45–79)

Table 2 presents the relationship between sex, age, and the sepsis continuum. The prevalence rates of sepsis and septic shock were higher in females (55% and 62.5%, respectively), but that of severe sepsis was higher in males (75%); however, the data showed no statistical significance (p = 0.1258). The associations of sepsis continuum with the median age of all patients (p = 0.7198), of males (p = 0.5621), and of females (p = 0.9525) were also not statistically significant.

**Table 2:** Relationship of sepsis continuum with sex and median age distributions. *The chi-squared test was used to test significance. ႵThe Kruskal–Wallis test was used to test significance.  IQR: interquartile range.

		Patients with sepsis (n = 80)	Patients with severe sepsis (n = 12)	Patients with septic shock (n = 8)	p-value
Sex	Male (%)	36 (45.0)	9 (75)	3 (37.5)	0.1258*
	Female (%)	44 (55.0)	3 (25)	5 (62.5)	
Median age in years (IQR)		59 (45–79)	59.50 (51–68)	59 (47–73)	0.7198^Ⴕ^
Median age of male in years (IQR)		58 (45–70)	63 (51–68)	57 (53–63)	0.5621^Ⴕ^
Median age of female in years (IQR)		59 (45–79)	59 (59–60)	59 (47–73)	0.9525^Ⴕ^

Table 3 lists the mean values of RBG and HbA1c across the sepsis continuum. The relationship of sepsis continuum with the mean RBG (p = 0.5745) and the mean HbA1c (p = 0.2784) was not statistically significant.

**Table 3:** Mean values of random blood glucose and HbA1c across the sepsis continuum. The Kruskal–Wallis test was used to test significance.

	Patients with sepsis (n = 80)	Patients with severe sepsis (n = 12)	Patients with septic shock (n = 8)	p-value
Mean RBG in mmol/L (SD)	17.60 (8.559)	20.38 (9.683)	15.98 (5.801)	0.5745
Mean percentage HbA1c (SD)	10.72 (2.605)	9.986 (2.516)	8.980 (1.499)	0.2784

Table 4 enumerates the suspected sources of infection across the sepsis continuum. Skin and soft tissue infections were the greatest, followed by respiratory infections and then urinary tract infections (sepsis: 73.5%, 20%, and 6.25%; severe sepsis: 91.66%, 8.33%, and 0%; septic shock: 50%, 37.5%, and 12.5%, respectively).

**Table 4:** Suspected sources of infection in patients who met the criteria for sepsis, severe sepsis, and septic shock.

Suspected source of sepsis	Patients with sepsis (n = 80)	Patients with severe sepsis (n = 12)	Patients with septic shock (n = 8)
Respiratory infection (%)	16 (20)	1 (8.33)	3 (37.5)
Urinary tract infection (%)	5 (6.25)	0	1 (12.5)
Skin/soft tissue infection (%)	59 (73.50)	11 (91.66)	4 (50)

As shown in Table 5, the mean white blood count was highest in the sepsis group (17.54), followed by the severe sepsis (16.67) and septic shock (15.99) groups.

**Table 5:** Average white blood count of patients who met the criteria for sepsis, severe sepsis, and septic shock.

	Patients with sepsis (n = 80)	Patients with severe sepsis (n = 12)	Patients with septic shock (n = 8)
Mean white blood cell count per 10^9^/L (SD)	17.54 (8.083)	16.67 (9.350)	15.99 (6.430)

As mentioned, only 11 patients used traditional medicine before hospital admission (Table 6), with the highest in the septic shock group (37.5%), followed by severe sepsis (37.5%) and sepsis (6.25%) groups.

**Table 6:** Total number of patients that sourced traditional medicine before hospital admission.

	Patients with sepsis (n = 80)	Patient with severe sepsis (n = 12)	Patients with septic shock (n = 8)
No. of patients (%)	5 (6.25)	3 (25)	3 (37.5)

## Discussion

Currently, the prevalence rates of sepsis in the IM wards in the Pacific Islands are still unreported. Nonetheless, this study investigated such prevalence in patients with T2DM admitted to the IM ward in Samoa along the sepsis continuum. We found that the sepsis category was the most common presentation, followed by severe sepsis and septic shock. This result may perhaps be credited to our convenience sampling scheme. However, it might also stem from physicians in Samoa readily identifying the initial changes in organ dysfunction (pulmonary, genitourinary, soft tissue, and skin) for people with sepsis that require admission to the IM ward.

Our study further revealed no significant association between the sepsis continuum and sex, possibly because the proportions of male and female participants were fairly equal. This result is consistent with a previous Irish study, which discovered that sex distribution has no significant difference between patients with and without sepsis in the emergency department (ED) [20]. However, our study did not compare sex distribution between patients with and without sepsis diagnosis along the sepsis continuum.

In addition, we found no significant relationship between median age and the sepsis continuum. This result may be explained by our strict inclusion criteria of patients aged 45-80 years; consequently, our median age across the sepsis continuum was extremely similar. This finding is not consistent with similar studies conducted in the United Kingdom, where age has been reported to be significantly different in ED patients manifesting sepsis [21,22]. These studies compared patients with sepsis across the sepsis continuum with those without sepsis to justify the observed differences. Future studies may wish to compare the age distributions of these two patient groups. They may also consider broadening the age range of participants to note any differences.

In the current study, considerably more individuals with septic shock used traditional medicine before being admitted to the IM ward than those with severe sepsis and sepsis. This result can possibly be related to healthcare barriers, such as the patient’s geographical location and poor access to satisfactory medical treatment, thereby resorting to traditional medicine instead. These barriers could also result in failure to meet patient’s medical needs within the appropriate time frame, ultimately leading to life-threatening complications. A study conducted in three Pacific Island countries, namely, Solomon Islands, Vanuatu, and Nauru, noted that traditional medicine is the most significant reason for amputation and delayed treatment of individuals with diabetic foot sepsis [23]. Prospective studies may be conducted to examine the cultural norms of using traditional medicine as an initial treatment for people with sepsis in Western Samoa.

With regard to study limitations, our study has a relatively small sample size, similar to other studies. A study in Ireland recorded 42 patients with sepsis in the ED [20]. However, studies in the United Kingdom generally used larger sample sizes [21,22]. All attempts were made to elucidate the prevalence of sepsis, severe sepsis, and septic shock in patients with T2DM admitted to the IM ward in Samoa. Given that participants were selected by convenience sampling using an inpatient registry book, a selection bias may have existed. This sampling scheme was chosen to simplify clinical file retrieval. Randomized sampling may be used in prospective studies. The recording of sepsis continuum diagnoses depended on this being explicitly written in individual patient files upon admission. Future studies may collect data according to clinically relevant sepsis criteria and its continuum and categorize patients accordingly. Additionally, no qSOFA scores could be obtained using the admission notes. The procalcitonin test was also unavailable at TTM hospital. Both the qSOFA score and the procalcitonin test aid in sepsis diagnosis [4]. Furthermore, data gathering depends on the legibility of written clinical notes. For illegible clinical notes, clinical personnel were consulted for confirmation. The most up-to-date sepsis definitions were also not used; our study used the previous definitions, which describe severe sepsis and the continuum of sepsis [2]. Prospective studies in Samoa can utilize the updated sepsis definitions.

In contrast, this study has a particular strength. It revealed the prevalence rate of sepsis continuum in patients admitted to the IM ward in Samoa. No similar studies have been conducted in the Pacific Islands, let alone Australia or New Zealand. Hence, our study findings may inspire future research.

## Conclusions

In patients with T2DM admitted to the IM ward within the sepsis continuum, the sepsis category was overwhelmingly the most frequent, followed by severe sepsis and septic shock. Most patients had poorly controlled blood glucose levels. Future infections can be prevented by maintaining well-controlled glycemic levels from the moment the patient is diagnosed with DM. Patients with T2DM in Samoa diagnosed with septic shock are likely to have used traditional medicine before admission. These findings are unique in the Pacific and Oceania, and further research is warranted.
